# Leukemia cells induce changes in human bone marrow stromal cells

**DOI:** 10.1186/1479-5876-11-298

**Published:** 2013-12-04

**Authors:** Sara Civini, Ping Jin, Jiaqiang Ren, Marianna Sabatino, Luciano Castiello, Jianjian Jin, Huan Wang, Yuanlong Zhao, Francesco Marincola, David Stroncek

**Affiliations:** 1 Cell Processing Section, Department of Transfusion Medicine, Clinical Center, National Institutes of Health (NIH), Building 10, Room 3C720, 9000 Rockville Pike, Bethesda, MD 20892-1184, USA; 2 Infectious Disease and Immunogenetics Section (IDIS), Department of Transfusion Medicine, Clinical Center, National Institutes of Health (NIH), Bethesda, MD 20892, USA; 3 Sidra Medical and Research Centre, Doha, Qatar

**Keywords:** Bone marrow stromal cells (BMSCs), Leukemia, Tumor microenvironment, Hematopoietic niche

## Abstract

**Background:**

Bone marrow stromal cells (BMSCs) are multipotent cells that support angiogenesis, wound healing, and immunomodulation. In the hematopoietic niche, they nurture hematopoietic cells, leukemia, tumors and metastasis. BMSCs secrete of a wide range of cytokines, growth factors and matrix proteins which contribute to the pro-tumorigenic marrow microenvironment. The inflammatory cytokines IFN-γ and TNF-α change the BMSC secretome and we hypothesized that factors produced by tumors or leukemia would also affect the BMSC secretome and investigated the interaction of leukemia cells with BMSCs.

**Methods:**

BMSCs from healthy subjects were co-cultured with three myeloid leukemia cell lines (TF-1, TF-1α and K562) using a trans-well system. Following co-culture, the BMSCs and leukemia cells were analyzed by global gene expression analysis and culture supernatants were analyzed for protein expression. As a control, CD34+ cells were also cocultured with BMSCs.

**Results:**

Co-culture induced leukemia cell gene expression changes in stem cell pluripotency, TGF-β signaling and carcinoma signaling pathways. BMSCs co-cultured with leukemia cells up-regulated a number of proinflammatory genes including IL-17 signaling-related genes and IL-8 and CCL2 levels were increased in co-culture supernatants. In contrast, purine metabolism, mTOR signaling and EIF2 signaling pathways genes were up-regulated in BMSCs co-cultured with CD34+ cells.

**Conclusions:**

BMSCs react to the presence of leukemia cells undergoing changes in the cytokine and chemokine secretion profiles. Thus, BMSCs and leukemia cells both contribute to the creation of a competitive niche more favorable for leukemia stem cells.

## Background

Acute myeloid leukemia (AML) is a clonal, malignant disorder. Treatment of AML is often complicated by disease propagation and relapse due to a small subset of cells called leukemia stem cells (LSC). LSC show a less mature phenotype compared with leukemia cells and they display a constitutive activation of factors such as NF-κB, Akt, and Wnt/β-Catenin which are involved in survival and self-renewal [1-3]. Leukemia stem cells are a heterogeneous population, which were first found among CD34^+^CD38^-^ populations, but they are also present among CD34^+^CD38^+^ and CD34^-^ cells [4]. Normal hematopoietic stem cells and LSCs reveal a high degree of similarity and although LSCs show increased expression of CD44, CD96, CD47 and the loss of CD90 expression, no unique LSC marker has yet been found [5-9].

In the hematopoietic niche, LSCs interact with bone marrow stromal cells (BMSCs) to create a microenvironment that is favorable for LSC survival [10,11]. The interactions between leukemia cells and the niche encompass membrane receptors and soluble factors. These factors include CXCR4/CXCL12 (SDF-1) signaling, which is involved in the homing, survival, and proliferation of leukemia cells in AML [12,13] and chronic myeloid leukemia (CML) [14]. It is also important to note that CD44 and VLA-4 receptors expressed by leukemia cells play a role in their adhesion to stromal cells in the niche and the consequent induction of anti-apoptotic effects that support leukemia cell survival [15,16].

BMSCs, which are also known as mesenchymal stromal cells or mesenchymal stem cells, are a multipotent population that plays an active role in the hematopoietic niche. They maintain hematopoietic stem cells (HSCs) dormant within the niche and they play a role in the release of activated HSCs [17-24]. These cells secrete a wide range of cytokines, growth factors and matrix proteins involved in the hematopoiesis and hematopoietic stem cells maintenance [25-30].

It has been shown that in chronic lymphocytic leukemia (CLL), BMSCs through cysteine-cysteine metabolism provide leukemia cells with the antioxidant species (GSH) and promote cell survival in oxidative stress conditions [31,32]. In multiple myeloma, BMSCs up-regulate the secretion of several factors (IL-6, IGF-1, VEGF, FGF, SDF-1 and TNFα) as a result of their direct interaction with myeloma cells through integrins and soluble factors produced by myeloma cells. This interaction of myeloma cells and BMSCs in turn promotes a pro-tumorigenic environment in which the survival, growth and drug resistance of multiple myeloma cells is guaranteed [33-35].

To further understand the interaction between BMSCs and leukemia stem cells in the bone marrow microenvironment, we selected three myeloid leukemia cell lines with different degrees of stemness and co-cultured them with BMSCs from healthy donors. We found that BMSCs responded to leukemia cells by up-regulating many pro-inflammatory and IL17-signaling related genes.

## Methods

### Study design

BMSCs from healthy donors were co-cultured with three different myeloid leukemia cell lines. AML cell lines TF-1 and TF-1α were selected because of their phenotype: CD34^+^/CD38^+^ and CD34^+^/CD38^-^, respectively; the TF-1α phenotype being less mature than the TF-1 phenotype. We also selected K562, a CD34- chronic myeloid leukemia cell line, as a third cell line of bone marrow origin. A 1-μm Transwell system (BD Biosciences, San Jose’, CA USA) was used to maintain the cultured BMSC and leukemia cell populations separate from each other. BMSCs were also co-cultured under the same conditions with CD34^+^ cells isolated from G-CSF-mobilized peripheral blood stem cells from healthy donors BMSCs, leukemia and CD34^+^ cells cultured alone (mono-cultures) were used as controls. Cells from both mono- and co-culture conditions were harvested at 4 h, 10 h, and 24 h. Supernatants were harvested at 48 h. Cells were analyzed for global gene expression profiles, culture media for selected cytokines and chemokines. These studies were approved by a NIH Institution Review Board.

### Bone marrow stromal cells, leukemia cell lines and hematopoietic stem cells

Passage 2 BMSCs from 4 healthy donor bone marrow aspirates were provided by the Bone Marrow Stromal Cell Transplant Center, NIH, Bethesda, Maryland. BMSCs were expanded and characterized as described in our previous work [29,36]. Briefly, cells from bone marrow aspirates were seeded in complete media (α - minimal essential medium (α-MEM), 2 mM glutamine, 10 μg/ml gentamicin and 20% fetal bovine serum) for 24 h, and the non-adherent cells were removed. The adherent cells were expanded until a 70-80% confluence was reached. Cells were sub-cultured until passage 4 and kept in complete media.

Leukemia cell lines were purchased from ATCC: TF-1 (#CRL2003) CD34^+^/CD38^+^, TF-1α (ATCC #CRL2451) CD34^+^/CD38^-^ and K562 (ATCC #CCL243) CD34^-^. The TF-1α and K562 cells were maintained in RPMI with 10% FBS. TF-1 cells were kept in RPMI with 10% FBS and 2 ng/μl of GM-CSF until use in co-culture experiments.

Human CD34^+^ hematopoietic stem cells from three different healthy donors were kindly provided by Dr J. Miller (NIH-NIDDK). Peripheral blood stem cells (PBSC) were collected by apheresis after 5 days of stimulation with G-CSF and CD34^+^ cells isolated from the PBSCs using CD34 antibodies conjugated to paramagnetic beads (ClinicMACS, Miltenyi Biotec Inc, Auburn, CA USA).

### Co-culture

Passage 4 BMSCs were seeded in the 6-well plates at a concentration of 5×10^4^ cells/well, in RPMI plus 10% FBS on day -1. At day 0, 1×10^6^ TF-1, TF-1α, K562 and CD34^+^ cells were seeded into the Transwell system. Mono-cultures of BMSCs, leukemia and CD34^+^ cells were seeded at the same above mentioned conditions as controls. Cells were harvested after 4 h, 10 h and 24 h, treated with 700 μl QIAzol (Qiagen, Valencia, CA USA) and were stored at -80°C until use. Supernatants collected after 48 h were stored immediately at -80°C. For some studies 1×10^6^ of the TF-1, TF-1α or K562 cells were cultured in direct contact with passage 4 BMSCs in 6 well plates.

### Total RNA purification, amplification, hybridization and slide processing

Total RNA from co-culture and control samples was purified using miRNA Easy Kit (Qiagen). The RNA concentration was measured using a Nano Drop ND-1000 Spectrophotometer (Nano Drop Technologies, Wilmington, DE, USA) and RNA quality was assessed with an Agilent 2100 Bioanalyzer (Agilent Technologies, Santa Clara, CA, USA).

RNA was amplified using an Agilent LowInput QuickAmp Labeling Kit Two color and subsequently co-hybridized with Universal Human Reference RNA (Stratagene, Santa Clara, CA, USA) on Agilent Chip Whole Human genome, 4x44k slides according to manufacturer’s protocol.

### Statistical and microarray data analysis

Images of the arrays were acquired using a microarray scanner Scan G2505B and image analysis was performed using Scan Control software version 9.5 (Agilent Technologies). The images were extracted using the Feature Extraction Software (Agilent Technologies). Partek Genomic Suite 6.4 (Partek Inc., St. Louis, MO, USA) was used for data analysis, visualization, identification of differentially expressed transcripts (unadjusted p-value < 0.05) and hierarchical cluster analysis. Ingenuity Pathway Analysis website (http://www.ingenuity.com, Ingenuity System Inc., Redwood City, CA, USA) was used for analysis of functional pathways. The microarray data used in this study have been deposited in National Center for Biotechnology Information Gene Expression Omnibus database (GSE45663).

### Quantitative real-time PCR analysis

To validate the results of the microarray analysis, we performed quantitative real-time PCR (RT-PCR) analysis on total RNA from co-cultures and controls using 18S rRNA as a housekeeping gene (Assay ID Hs99999901_s1, Applied Biosystem-Life Technologies). Genes used for validation were selected from those most up-regulated in co-cultured cells compared to mono-culture controls: *IL8, CCL2, ICAM1* and *IL1B*. Gene expression data were quantified with TaqMan Gene Expression Assay for each of the above mentioned genes (Assay IDs: Hs99999034_m1, Hs00234140_m1, Hs00164932_m1, Hs01555410_m1, respectively, Life Technologies), according to manufacturer’s protocol. For each sample, relative gene expression level was normalized to 18S rRNA and determined by the 2^-ΔΔCt^ method. The reaction was performed using ABI Prism (BD Biosystem, Life Technologies). The resulting data were analyzed by SDS and RQ software (Applied Biosystem). The results were shown as the relative co-culture mRNA level to mono-culture control mRNA for the selected genes.

### Proteome profiles (Human Cytokine Array Panel A array kit)

Supernatants collected from co-cultured and control cells, after 48 h of culture, were thawed and immediately analyzed using the Human Cytokine Array Panel A array Kit (R&D System, Inc., Minneapolis, MN USA) following the manufacturer’s protocol. Briefly, 1 ml of supernatant was incubated for 1 h with 15 μl of human cytokine detection antibody cocktail. The suspension was incubated with the provided membrane at 4°C for 30 h and treated with the secondary antibody (Streptavidin-HRP) for 1 h at room temperature. The membrane was exposed to chemiluminescence reagents SuperSignal West Pico Chemiluminescent Substrate (Thermo Scientific, Rockford, IL, USA). After exposing the membranes for 30 min to X-ray film, the resulting film was scanned and the pixels were counted and analyzed with ImageJ software (CIT, NIH USA). The mean pixel density for each spot was calculated by background subtraction and each value was normalized by internal positive controls. Each sample was tested in duplicate.

### ELISA analysis

Levels of IL-8, and CCL2 in the supernatants from mono and co-cultured samples were measured with enzyme-linked immune adsorbent assays (Quantikine human kits; R&D System) following the manufacturer’s instructions using Victor^3^V ELISA reader (Perkin Elmer, Waltham, MA, USA). Minimal detectable levels were: IL-8, 3.5 pg/ml and CCL2, 1.7 pg/ml.

## Results

### Global gene expression analysis of BMSCs co-cultured with leukemia cells reveals up-regulation of IL-17 signaling-related genes

To study the effects of leukemia cells on BMSCs, we co-cultured BMSCs from healthy donors with three different leukemia cell lines, TF-1α, TF-1 and K562, that were selected according to their phenotypes: CD34^+^/CD38^-^, CD34^+^/CD38^+^ and CD34^-^, respectively. The BMSCs and leukemia cells were co-cultured in transwells without physic contact. The cells were harvested at 4 h, 10 h and 24 h and total RNA was extracted. The gene expression profiles of BMSC mono-cultures and BMSCs co-cultured with the three leukemia cell lines were analyzed.

**Figure 1:**
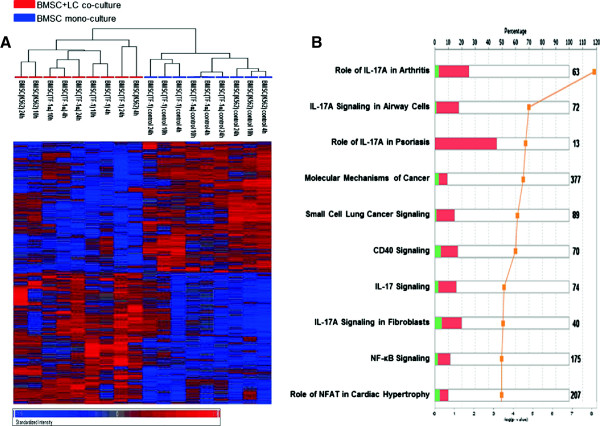
**Gene expression analysis of BMSCs co-cultured with leukemia cells compared with BMSC mono-cultures shows changes in IL-17 signaling-related genes. (A)** Hierarchical clustering analysis of 1540 differentially expressed genes in BMSCs co-cultured in transwells with three leukemia cell lines (TF-1, TF-1α and K562) compared with BMSC mono-cultures (control) using Partek Genomic Suite program (ANOVA test with unadjusted p-value < 0.05). The displayed colors represent the fold changes where shades of red and blue indicate up- and down-regulation respectively. The color key for the sample labels is on the top left. **(B)** Ingenuity Pathway Analysis (IPA) of the 1540 differentially expressed BMSC genes. Numerical symbols at the right side of each bar indicate the total number of genes composing the pathway. The bars indicate the percentage of up- (red bar) and down-regulated (green bar) genes in each pathway, while the orange line indicates minus-log transformed p-value. The top 10 canonical pathways are shown.

The overall comparison between mono- and co-culture BMSCs revealed that 1540 BMSC genes were differentially expressed (p-value <0.05). Supervised hierarchical clustering analysis of those genes clearly separated the BMSC samples into two groups: co-cultured and mono-cultured BMSCs (Figure 1A). We found that *IL8, CXCL1, IL1B, CXCL3, CCL2, CXCL3, CXCL2* and *ICAM1* genes, all of which are known to be involved in the acute inflammatory response, were the most up-regulated genes in BMSCs co-cultured with leukemia cells (Table 1). Ingenuity Pathway Analysis (IPA) of the differentially expressed genes revealed that the most over-represented canonical pathways were the IL-17 signaling, CD40 signaling and NFκB signaling pathways (Figure 1B). We also compared the microarray data from the different time points and we found that most of the changes in the BMSC gene expression profiles occurred within 4 h (data not shown).

**Table 1:** Change in expression of BMSC genes during co-culture with leukemia cells

**Up-regulated genes**^**a**^		**Down-regulated genes**^**a**^	
**Gene symbol**	**Fold change**	**Genes symbol**	**Fold change**
*IL8*	19.9	*ANGPT2*	-3.4
*CXCL1*	19.7	*CSRNP3*	-2.8
*IL1B*	8.8	*TMEM26*	-2.7
*CXCL3*	7.6	*GPR85*	-2.6
*CCL2*	7.3	*F2RL2*	-2.6
*CXCL2*	6.3	*FOSB*	-2.5
*ICAM1*	5.2	*PROCK3*	-2.4
*SERPINB2*	4.6	*AP4E1*	-2.4
*PTGS2*	4.5	*EVC*	-2.3

^a^Comparison of co-cultured and mono-cultured BMSCs (p-value < 0.05).

The genes with the greatest fold change are shown.

Next, we checked if BMSCs responded differently to the three different leukemia cell lines. The microarray data were analyzed separately for BMSCs co-cultured with the three different leukemia cell lines and we found that BMSCs reacted somewhat differently when co-cultured with each of the three leukemia cell lines. Using Partek Genomic Suite, we found that the number of differentially expressed genes in BMSCs co-cultured with TF-1, TF-1α and K562 compared with BMSC mono-cultures were 1775, 1375 and 1738 respectively. The genes *IL8, CCL2, CXCL1, IL1B* and *ICAM1* were among the most up-regulated genes in BMSCs co-cultured with both TF-1 and K562 although with significantly different fold changes (Table 2). In contrast, analysis of BMSCs co-cultured with TF-1α revealed a different signature with a mild up-regulation of *IRF8* and *CADHERIN7* and a down-regulation of *COL3A1* (Table 2). Ingenuity pathway analysis of the three separate sets of BMSC differentially expressed genes revealed that the top canonical pathways involved were IL-17 signaling, CD40 signaling and IL-6 signaling in BMSCs co-cultured with TF-1 and K562, while *Rac* signaling, actin cytoskeleton signaling, growth hormone signaling and death receptor signaling were among the most over-represented canonical pathways in BMSC co-cultured with TF-1α (Table 2).

**Table 2:** **Change in expression of BMSC genes during co-culture with 3 different leukemia cell lines and with CD34**^**+**^**cells**

**Class comparison**^**a**^	**Up-regulated genes**		**Down-regulated genes**		**Most over-represented canonical pathways**^**b**^
	**Gene symbol**	**Fold change**	**Genes symbol**	**Fold change**	
**BMSCs co-cultured with TF-1**	*CXCL1*	338.3	*CYP3A4*	-5.9	*Role of IL-17A in arthritis*
	*IL8*	168.8	*PLEKHH2*	-4.2	*Role of IL-17 F in allergic Inflammatory airway Diseases*
	*CXCL3*	59.4	*CCNF*	-4.0	*Role of IL-17A in Psioriasis*
	*CXCL2*	46.6	*CYP3A5*	-3.9	*IL-17A signaling in airway cells*
	*PTGS2*	32.8	*RBM5*	-3.8	
	*IL1B*	26.6	*BMP4*	-3.8	
	*IL1A*	26.5	*ANGPT2*	-3.7	
	*IL1B*	23.1	*GZMB*	-3.5	
	*ICAM1*	21.7	*DAB2IP*	-3.5	
	*CCL2*	19.3	*CYP3A7*	-3.5	
**BMSCs co-cultured with TF-1α**	*IRF8*	4.2	*Col3a1*	-14.4	*Rac signaling*
	*SLC30A4*	4.0	*Gria1*	-6.6	*D-myo-inositol(1,4,5) triphosphate biosynthesis*
	*CDH7*	3.2	*ASB5*	-5.5	
	*DZANK1*	3.0	*ABCC9*	-5.2	*Actin cytoskeleton signaling*
	*GDF2*	3.0	*DBIL5P*	-4.2	*Growth hormone signaling*
	*NPTX1*	2.9	*CALB2*	-3.5	*Death receptor signaling*
	*ATPBD4*	2.9	*TMEM242*	-3.5	
	*UNC5C*	2.9	*EIF4EBP2*	-3.4	
	*YOD1*	2.9		-3.1	
	*CCL28*	2.8	*MLL2*	-3.0	
**BMSCs co-cultured with K562**	*IL8*	17.4	*SPOCK3*	-7.1	*Role of IL-17A in arthritis*
	*CCL2*	12.6	*KANK4*	-6.5	*Hepatic fibrosis/stellate cell activation*
	*PTPRR*	11.9	*PRSS35*	-6.5	*VDR/RXR activation*
	*IL1B*	11.6	*GPR85*	-5.5	*CD40 signaling*
	*CXCL1*	9.2	*FGF7*	-4.7	*IL-6 signaling*
	*ICAM1*	8.4	*SLC8A1*	-4.6	
	*SLC25A21*	7.6	*AP4E1*	-4.6	
	*SERPINB2*	7.5	*ABCC9*	-4.2	
	*SLC44A4*	6.1	*C17orf28*	-4.2	
	*FAM167A*	5.0	*FLT1*	-4.1	
**BMSCs co-cultured with CD34**^**+**^**cells**	*SERPINB2*	3.5	*UGT2B10*	-2.9	*Purine metabolism*
	*IL1B*	3.4	*METTL3*	-2.7	*Estrogen receptor signaling*
	*RTP3*	2.8	*SFP2*	-2.6	*mTOR signaling*
	*ZBED2*	2.7	*C5AR1*	-2.5	*EIF2 signaling*
	*CCL7*	2.6	*ISL2*	-2.5	*Aminosugars metabolism*
	*ERMN*	2.6	*KLRC3*	-2.4	
	*IL8*	2.6	*EFHD1*	-2.3	
	*NKAPL*	2.5	*RFX4*	-2.3	
	*TSLP*	2.4	*KANK4*	-2.2	

^**a**^Comparison of co-cultured and mono-cultured BMSCs (p-value <0.05).

^b^Ingenuity pathway analysis (IPA).

The genes with the greatest fold change are shown.

**Figure 2:**
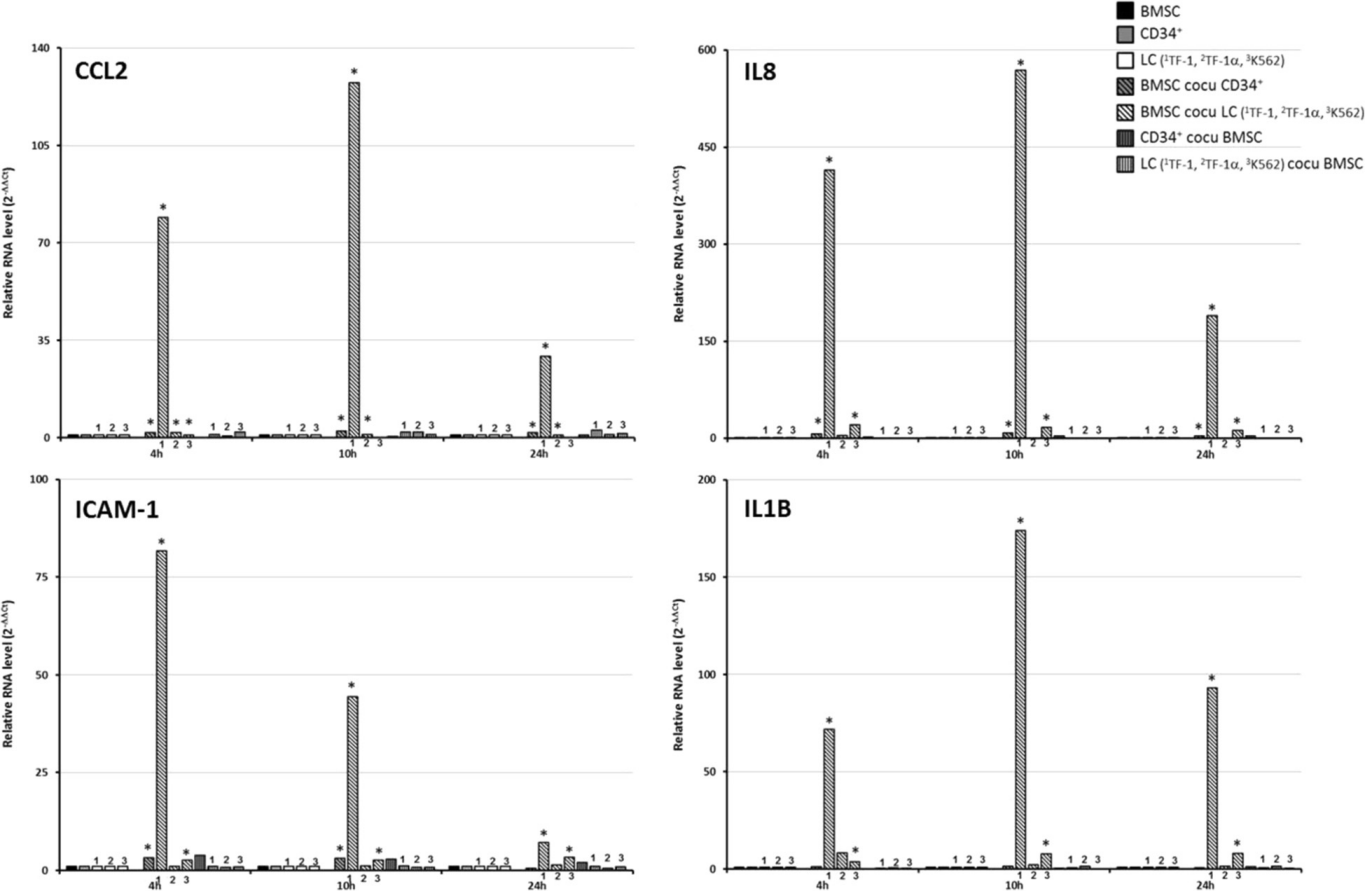
**The expression of IL-17 signaling-related genes increase in BMSCs co-cultured with leukemia cells.** Quantitative RT-PCR was performed to quantify the expression levels of *CCL2*, *ICAM1*, *IL8* and *IL1B* in BMSCs (black column), CD34+ cells (grey bars) and TF-1 (1), TF-1a(2) and K562(3) leukemia cell (LC) (white bars) mono-cultures, BMSCs co-cultured in transwells with leukemia cell lines (black and white stripped column) and BMSCs co-cultured in transwells with CD34+ cells (grey and black stripped column). The RNA levels were shown as 2^-ΔΔCt^ method. Sample key legend is at the top right. * p-value < 0.05

To validate the microarray data, we performed quantitative RT-PCR analysis. The RT-PCR results confirmed the greater expression of *CCL2, ICAM1, IL8* and *IL1B* in BMSCs co-cultured with leukemia cells compared with BMSC mono-cultures (Figure 2).

To study the effects of BMSCs on leukemia cells, the gene expression profiles of TF-1, TF-1α and K562 leukemia cells alone and co-cultured with BMSCs were analyzed by microarrays. The microarray data were analyzed using Partek Genomic Suite and the analysis revealed that 1138, 1119 and 943 genes were differentially expressed (p-value <0.05) in TF-1, TF-1α and K562 cells co-cultured with BMSCs compared with the respective leukemia cell mono-cultures. Among the most up-regulated genes were *RGS1*, *FAM69A*, *Skg1* and *SOCSs*, although their fold change in expression was <7. Ingenuity pathway analysis (IPA) of the differentially expressed genes revealed that the most represented canonical pathways were stem cells pluripotency, TGF-β signaling and carcinoma signaling (Table 3).

**Table 3:** **Change in expression of leukemia and CD34**^**+**^**cell genes during co-cultured with BMSCs**

**Class comparison**^**1**^	**Up-regulated genes**		**Down-regulated genes**		**Most over-represented canonical pathways**^**2**^
	**Gene symbol**	**Fold change**	**Genes symbol**	**Fold change**	
**TF-1 cells co-cultured with BMSCs**	*RGS1*	6.04	*HARS2*	-4.44	*Role of NANOG in stem cells pluripotency*
	*TST*	4.93	*CBL*	-4.14	*CNTF signaling*
	*CD93*	4.46	*WDR31*	-3.56	*Growth hormone signaling*
	*AZGP1*	4.34	*PIGR*	-3.37	*IGF-1 signaling*
	*CD200R1*	3.95	*SLC12A1*	-3.19	*Stem cells pluripotency*
	*CTNND!*	3.37	*CTTNBP2*	-3.11	
	*ASAP2*	3.28	*C6orf105*	-2.97	
	*CLEC4M*	3.23	*ATP2B4*	-2.79	
	*CACNA1F*	3.20	*SLC16A6*	-2.75	
	*SOCS7*	3.19	*SLC38A10*	-2.74	
**TF-1α cells co-cultured with BMSCs**	*FAM69A*	4.22	*ADPRH*	-7.62	*Role of macrophages in rheumatoid arthritis*
	*LIM2*	3.96	*SIGLEC12*	-6.85	*Axonal guidance signaling*
	*KCNMB2*	3.60	*XIRP2*	-5.00	*Basal cell carcinoma signaling*
	*C1QL1*	3.28	*KRT2*	-4.42	*G-protein coupled receptor signaling*
	*TULP1*	3.27	*GPR180*	-3.91	*ERK/MAPK signaling*
	*DIS3*	3.22	*KLHL1*	-3.87	
	*IGFBP4*	3.22	*GALNT4*	-3.55	
	*SSPN*	3.19	*PDGFC*	-3.50	
	*ATP11A*	3.18	*PFN4*	-3.32	
	*SLC25A2*	3.07	*GPR37L1*	-3.27	
**K562 cells co-cultured with BMSCs**	*SKG1*	3.66	*EPB42*	-5.78	*Role of Oct4 in stem cell pluripotency*
	*KLARN*	3.41	*EPX*	-5.00	*Antigen presentation pathway*
	*CNN1*	3.31	*BPIFA1*	-4.38	*TGF-β signaling*
	*AFF3*	3.11	*LEPR*	-3.93	*Regulation of IL-2 expression in T lymphocyte*
	*SKIL*	3.04	*ANKRD11*	-3.24	*Semaphorin signaling in neurons*
	*SLC2A12*	2.93	*HIST1H1B*	-3.18	
	*ATXN7*	2.80	*SORBS1*	-3.15	
	*LEFTY1*	2.52	*CYP4A11*	-3.10	
	*DMBT1*	2.41	*GRWD1*	-3.07	
	*CELF2*	2.34	*CNOT4*	-2.91	
**CD34**^**+**^**cells co-cultured with BMSCs**	*SOCS3*	14.45	*HMOX1*	-3.26	*cAMP-mediated signaling*
	*REN*	11.56	*C18orf1*	-3.17	*VDR/RXR activation*
	*ECEL1*	7.70	*POU2F2*	-3.09	*Cardiac β-adrenergic signaling*
	*ATP6V0A4*	7.63	*PLD4*	-3.07	*Dopamine-DARPP32 feedback in cAMP signaling*
	*NR4A3*	6.83	*GPR44*	-3.06	
	*GEM*	6.79	*RXFP1*	-2.93	
	*HAS1*	6.64	*TNFSF14*	-2.92	
	*KIAA1199*	6.48	*ENC1*	-2.78	
	*PDLIM3*	6.22	*CST6*	-2.68	
	*CXCL6*	5.74	*CYTH4*	-2.62	

^1^Comparison of leukemia or CD34+ cells co-cultured with BMSCs compared with mono-cultured leukemia or CD34+ cells (p-value < 0.05, FDR < 0.01).

^2^Ingenuity Pathway Analysis (IPA).

**Figure 3:**
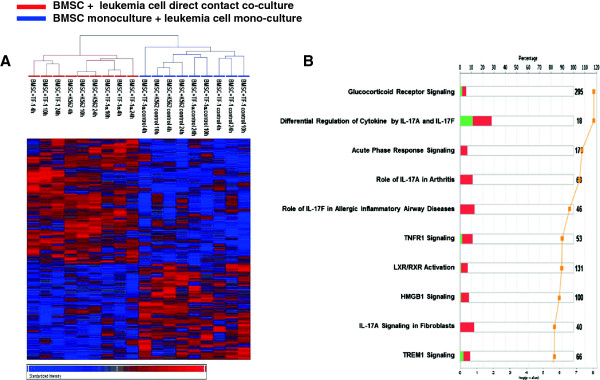
**Gene expression analysis of BMSCs co-cultured in direct contact with leukemia cells shows changes in IL-17 signaling-related genes.** BMSCs from three healthy donors were co-cultured in direct contact with the three different leukemia cell lines (TF-1, TF-1α and K562). The cells were harvested at 4 h, 10 h and 24 h and total RNA were extracted. The total RNA from BMSC mono-cultures was mixed with the total RNA from TF-1, TF-1α or K562 cell mono-cultures and the resulting three mixed total RNA samples were used as a “mono-culture” control and were subjected to gene expression analysis. The RNA from BMSCs and leukemia cells co-cultured in direct contact was extracted and gene expression was analyzed. Analysis with Partek Genomic Suite revealed that 544 genes that were differently expressed between the BMSCs and leukemia cell direct contact co-culture samples and the mono-culture controls (p-value < 0.05, FDR < 0.01). **Panel A**; Hierarchical clustering analysis of 544 differentially expressed genes in BMSCs and leukemia cell direct contact co-cultre samples compared with the mono-cultures (control) using Partek Genomic Suite program (ANOVA test with unadjusted p-value < 0.05). The displayed colors represent the fold changes where shades of red and blue indicate up- and down-regulation respectively. The color key for the sample labels is on the top left. **Panel B**. Ingenuity Pathway Analysis (IPA) of the 544 differentially expressed genes. Numerical symbols at the right side of each bar indicate the total number of genes composing the pathway. The bars indicate the percentage of up- (red bar) and down-regulated (green bar) genes in each pathway, while the orange line indicates minus-log transformed p-value. The top 10 canonical pathways are shown.

Next, we studied the effects of leukemia cells on BMSCs co-cultured in direct contact. BMSCs from three healthy donors were co-cultured with the three different leukemia cell lines in direct contact. The cells were harvested at 4 h, 10 h and 24 h and total RNA was extracted. The total RNA from BMSC mono-cultures was mixed with the total RNA from TF-1, TF-1α or K562 cell mono-cultures and the resulting three mixed total RNA samples were used as a “mono-culture” control in the gene expression profiling analysis. The RNA from BMSCs co-cultured with the TF-1, TF-1a and K562 cells were extracted and the gene expression profiles were analyzed by microarrays. The analysis of microarray data using Partek Genomic Suite revealed that 544 genes were differentially expressed between co-cultured and mono-cultured control cells (p-value <0.05, FDR <0.01). Hierarchical clustering analysis of these genes clearly separated the samples into two groups: co-cultures and mono-cultures (Figure 3A). The results were similar to the analysis of BMSCs co-cultured in transwells with the leukemia cells. We found that *CXCL1*, *CXCL6*, *TEP1*, *IL8*, *CCL2* and *PTGS2* genes were the most up-regulated genes in BMSCs co-cultured in the direct contact with leukemia cells. Ingenuity Pathway Analysis of the differentially expressed genes revealed that the top canonical pathways involved were the glucocorticoid receptor signaling, IL-17 signaling and acute phase response signaling (Figure 3B).

### Gene expression analysis of BMSCs co-cultured with CD34^+^ cells revealed changes in metabolism related genes

**Figure 4:**
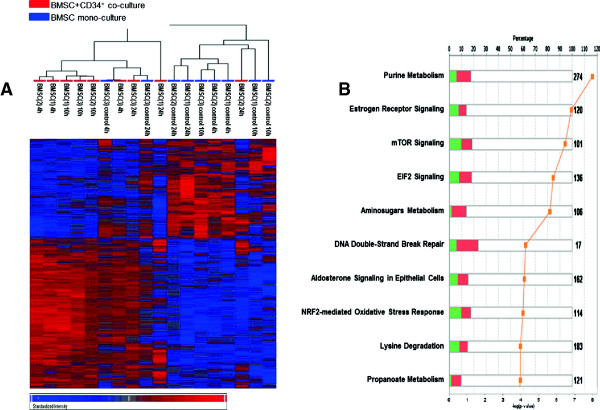
**Gene expression analysis of BMSCs co-cultured with CD34**^**+ **^**cells compared with BMSC mono-cultures shows changes in metabolism related genes. (A)** Hierarchical cluster analysis of 4904 differentially expressed genes in BMSCs co-cultured in transwells with CD34^+^ cells from three healthy donors compared with BMSC mono-cultures (control) using Partek Genomic Suite program (ANOVA test with unadjusted p-value < 0.05). The displayed colors represent the fold changes with the shades of red and blue indicating up- and down-regulation respectively. The color key for the sample labels is on the top left. **(B)** Ingenuity Pathway Analysis (IPA) of the 4904 differentially expressed BMSC genes. Numerical symbols at the right side of each bar indicate the total number of genes in each pathway. The bars indicate the percentage of up- (red bar) and down-regulated (green bar) genes in each pathway, while the orange line indicates minus-log transformed p-value. The top 10 canonical pathways are shown.

To evaluate whether the observed BMSC gene induction was specifically induced by leukemia cells, BMSCs were co-cultured in transwells with CD34^+^ cells from healthy donors*.* The BMSCs were harvested at 4 h, 10 h and 24 h and total RNA was extracted. The gene expression profiles of BMSC mono-cultures and co-cultured with the CD34^+^ cells were analyzed by microarrays. Analysis of the microarray data revealed that 4904 genes were differentially expressed between the two groups (p-value <0.05). Hierarchical clustering analysis of those genes separated the BMSCs into two groups but the separation between co-cultured and mono-cultured cells was not perfect. One group consisted of 8 co-cultured samples and 2 mono-cultures; the second group consisted of 7 mono-cultured samples and 1 co-cultured sample (Figure 4A). We found that the most up-regulated genes in BMSCs co-cultured with CD34^+^ cells compared with BMSC mono-cultures were *SERPINB2, IL1B, RTP3, CCL7* and *IL8* (Table 2). Ingenuity pathway analysis (IPA) revealed that the top canonical pathways involved were the purine metabolism, mTOR signaling and EIF2 signaling (Figure 4B). To validate the microarrays data, we performed a quantitative RT-PCR analysis which confirmed the greater expression of *IL8* in BMSCs co-cultured with CD34^+^ cells compared with BMSC mono-cultures (Figure 2).

Similarly to what was done with the leukemia cell lines, to study the effects of BMSCs on CD34^+^ cells, gene expression profiles from CD34^+^ cell mono-cultures and co-cultures with BMSCs were analyzed by microarrays. We found that 2075 genes were differentially expressed (p-value <0.05) in CD34^+^ cell co-cultures compared with mono-cultures. Among the most up-regulated genes were *SOCS3, REN* and *CXCL6*; all with a fold change >5. Ingenuity pathway analysis of the differentially expressed genes revealed that the most represented canonical pathways were cAMP-mediated signaling, VDR/RXR activation and cardiac β-adrenergic signaling (Table 3).

### CCL2 and IL-8 are increased in supernatants from BMSCs co-cultured with leukemia cells

**Figure 5:**
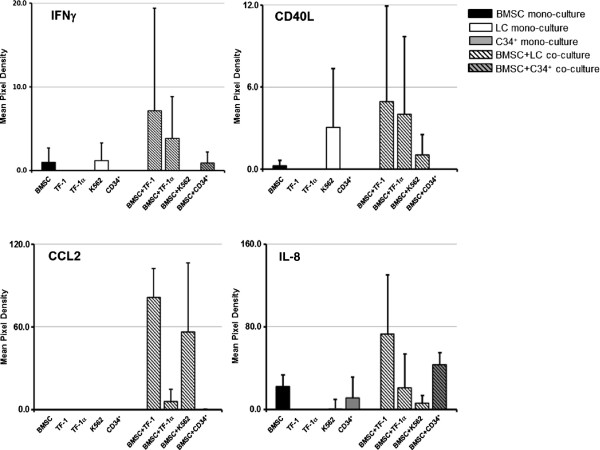
**IL-17 signaling-related cytokine levels are greater in supernatants from BMSCs co-cultured with leukemia cells.** Supernatants from the cultures were harvested after 48 hours and tested for soluble factors using an immunoblotting assay. The supernatants were from BMSC mono-cultures (black bars), TF1, TF-1α and K562 leukemia cell mono-cultures (white bars), CD34^+^ cells mono-cultures (grey bars), BMSCs co-cultured in transwells with leukemia cells (black and white stripped bars) and BMSCs co-cultured with CD34^+^ cells (black and grey stripped bars). The relative expression of selected cytokines was measured using array protein panel A (R&D System). The relative concentrations of IFN-γ, CD40L, CCL2 and IL-8 were calculated as a mean pixel density after normalization with positive control and background subtraction. The sample labeling legend is at the top right. The histograms represent results of 3 experiments with BMSCs from three different healthy donors.

**Figure 6:**
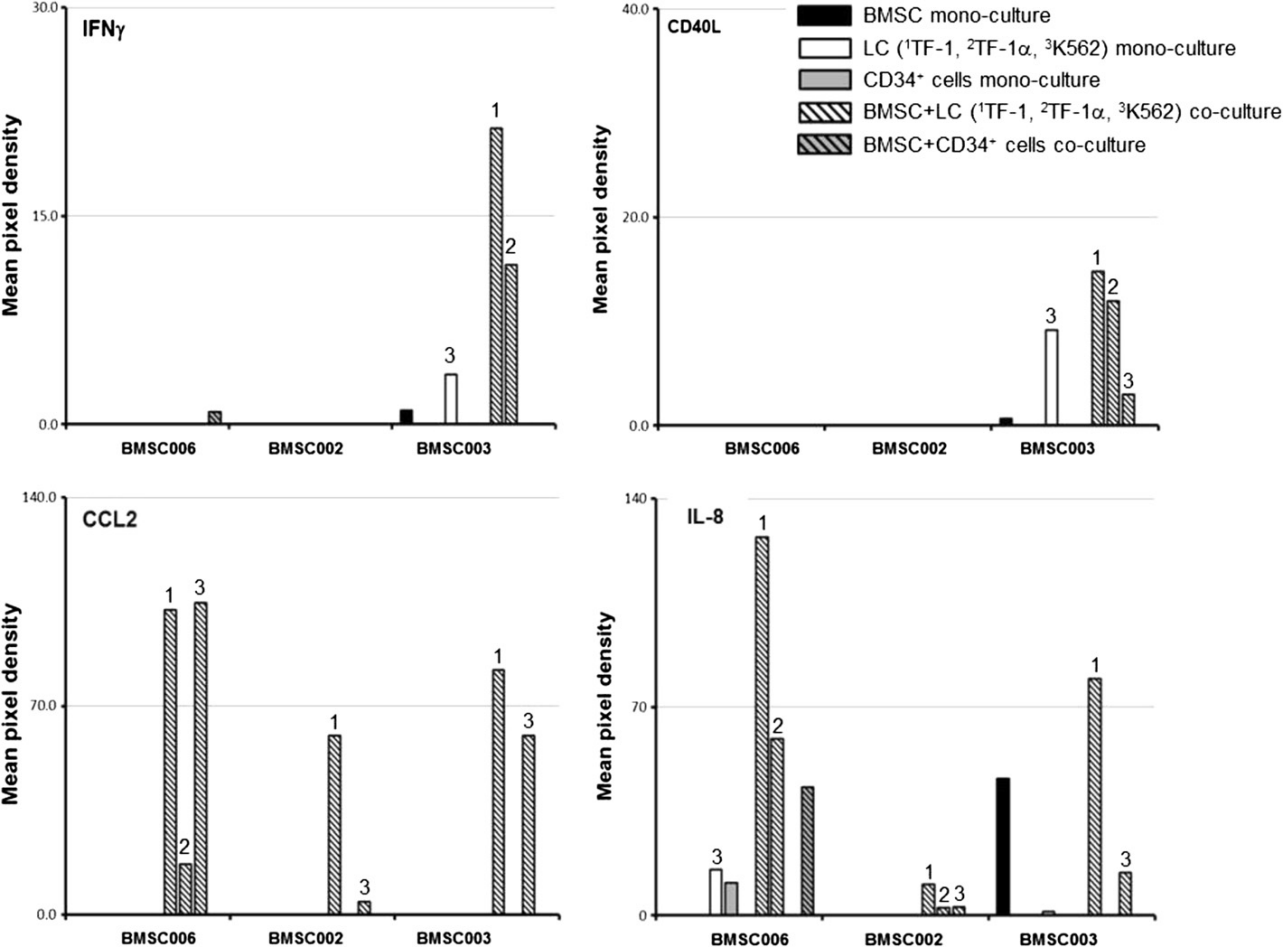
**IL-17 signaling-related cytokine levels increase in supernatants from BMSCs co-cultured with leukemia cells: comparison of the effects of BMSCs from 3 different donors.** BMSC mono-culture (black bars), TF1 (1), TF-1a (2) and K562 (3) leukemia cell (LC) mono-culture (white bars) and CD34^+^ cell mono-culture (grey bars), BMSC/leukemia cell transwell co-culture (black and white stripped bars) and BMSC/CD34+ cell transwell co-culture (black and grey stripped bars) supernatants were harvested after 48 h. The relative expression of selected cytokines was measured using array protein panel A (R&D System) immunobloting assay. The relative amounts of IFN-γ, CD40L, CCL2 and IL-8 were calculated as a mean pixel density after normalization with positive control and background subtraction. The sample labeling legend is at the top right. The results of 3 experiments with BMSCs from three healthy donors (BMSC006, BMSC002 and BMSC003) are shown.

Gene expression analysis revealed that most of the genes up-regulated in BMSCs co-cultured with leukemia cells were involved in IL-17 signaling. To assess the factors produced by co-cultured cells, we screened the supernatants from co-culture and mono-culture samples at 48 h for cytokine production by R&D Human Cytokine panel A. We chose this panel because among the 36 cytokines in the panel were CXCL1, sICAM-1, IL-1B, IL-8, CCL2 and Serpin E1 all of which were found to be up-regulated at the gene level in co-cultured BMSCs. Moreover, with panel A we were able to measure the relative levels of IFNγ, IL-6 and IL-23 which are IL-17 signaling-related cytokines. However, most of the 36 cytokines in the panel were undetectable in our samples and the levels of cytokines CXCL1, ICAM-1, IL-23, IL-6, MIF and Serpin E1 were not significantly changed between co-culture and mono-culture conditions (data not shown). However, the levels of CCL2 and IL-8 were greater in supernatants from BMSCs co-cultured with leukemia cells (Figure 5), but the results were variable among BMSCs from different subjects. The levels of IFNγ and CD40L were greater in co-culture compared with mono-culture supernatants, but the difference was not statistically significant. The analysis of cytokines in the supernatant of cultured BMSCs and leukemia cells was performed in three series of experiments with BMSCs from three healthy donors, (BMSC002, BMSC003 and BMSC006) and we found different responses among the different BMSC donors. We found increased levels of IFNγ and CD40L only in the supernatants from BMSC003 co-cultured with TF-1 and TF1α. The levels of IL-8 were increased in the supernatants from BMSC003, BMSC006 and, to a lesser extent, in BMSC002. The level of CCL2 was measurable only in supernatants from BMSC003, BMSC002 and BMSC006 co-cultured with TF-1 and K562 leukemia cells and in the supernatant from BMSC006 co-cultured with TF-1α (Figure 6).

**Figure 7:**
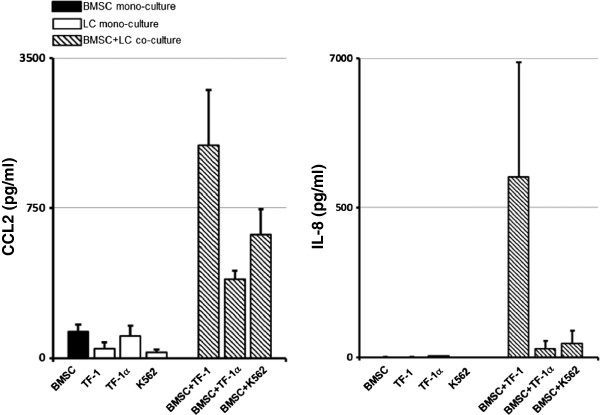
**CCL2 and IL-8 supernatant levels are greater in BMSCs co-cultured with leukemia cells.** BMSC mono-culture (black bars), TF1, TF-1α and K562 leukemia cell mono-culture (white bars) and BMSC/leukemia cell transwell co-cultures (black and white stripped bars) supernatants were harvested after 48 h. The concentration (pg/ml) of CCL2 and IL-8 was measured using an ELISA assay. The sample labeling legend is at the top left. The figures represent the results of 3 experiments with BMSCs obtained from three different healthy donors.

**Figure 8:**
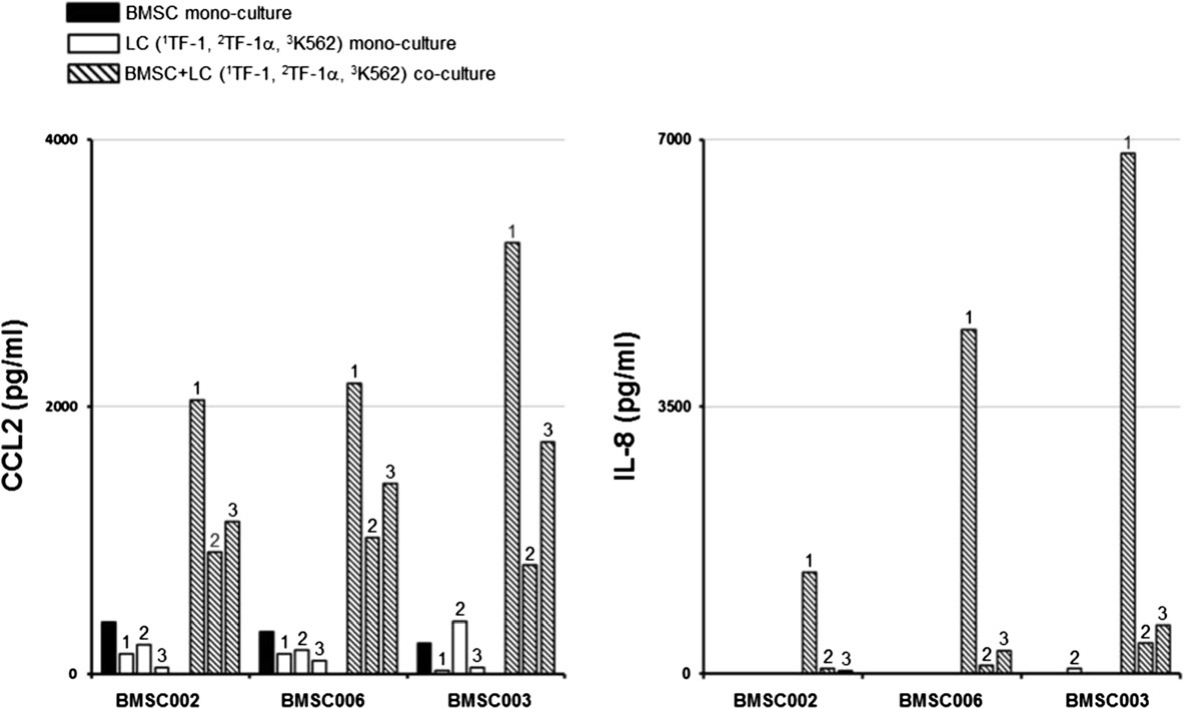
**CCL2 and IL-8 levels increase in supernatants from BMSCs co-cultured with leukemia cells: comparison of the effects of BMSCs from 3 different donors.** BMSC mono-culture (black bars), TF-1 (1), TF-1a (2) and K562 (3) leukemia cell mono-culture (white bars) and BMSC/leukemia cell transwell co-culture (black and white stripped bars) supernatants were harvested after 48 h. The amount (pg/ml) of CCL2 and IL-8 was measured by an ELISA assay. The sample labeling legend is at the top left. The results of 3 experiments with BMSCs from three healthy donors (BMSC006, BMSC002 and BMSC003) are shown.

To confirm the increased levels of CCL2 and IL-8 in the supernatants from BMSCs co-cultured with leukemia cells at 48 h, we measured the levels of the two cytokines using ELISA assays. We co-cultured BMSCs from 3 different healthy donors with TF-1, TF-1α and K562 leukemia cells and harvested the supernatants from the co-cultures and mono-cultures at 48 h. We found that the concentration of CCL2 in BMSCs and TF-1, TF-1α and K562 mono-cultures was 310.9 ± 77.3 pg/ml, 108.3 ± 74 pg/ml, 262 ± 112 pg/ml and 63.6 ± 30.7 pg/ml respectively. The concentration of CCL2 increased significantly in the supernatant of BMSCs co-cultured with TF-1, TF-1α and K562 (2482 ± 647 pg/ml, 915.3 ± 103 pg/ml and 1434 ± 298 pg/ml respectively) (Figure 7). The concentration of IL-8 in BMSC monocultures was <3.5 pg/ml for two of the donors and was 9.8 pg/ml in the third donor. The concentration of the secreted IL-8 was <3.5 pg/ml in the supernatants from TF-1 and K562 mono-cultures, but was higher (68.4 pg/ml) in the supernatants from TF-1α mono-cultures. The concentration of IL-8 increased in BMSCs co-cultured with TF-1, TF-1α and K562 (4216 ± 2760 pg/ml, 194 ± 180 pg/ml and 326.2 ± 300 pg/ml respectively) (Figures 7 and 8).

## Discussion

The purpose of our study was to investigate the effect of the leukemia microenvironment on bone marrow stromal cells. BMSCs support both normal and abnormal hematopoiesis. In leukemia microenvironment they play an important and complex role: BMSCs promote AML cell growth and drug resistance [37] via IL-6 secretion, JAK/STAT pathway activation [38] and by activating pro-survival pathways via integrin-linked kinases [39]. In chronic myeloid leukemia, BMSCs stabilize leukemia cells by promoting the clustering of CXCR4 in the lipid rafts and facilitating the migration of leukemia cells in the bone marrow [14]. BMSCs via the secretion of soluble factors also inhibit drug-induced apoptosis of AML [40] and myeloma cells [41]. It has been found that conditioned media from BMSCs cultured alone had no effect on myeloma cells, but soluble factors produced by BMSCs in contact with myeloma induced some anti-apoptotic properties suggesting a dynamic interaction between BMSCs and myeloma [41].

Our studies found a similar dynamic relationship between BMSCs and leukemia cells. We confirmed that BMSCs affect leukemia cells and found that leukemia cells change the profile of cytokines produced by BMSCs to a proinflammatory signature. These changes did not require direct contact between BMSCs and leukemia cells; they were mediated by soluble factors. In an *in vitro* co-culture model, BMSCs responded to the presence of leukemia cells undergoing changes in gene expression and cytokine release. After co-culture with leukemia cells 1540 BMSC genes were differentially expressed. The most up-regulated genes were involved in the acute inflammatory response and in the IL-17, CD40 and NFκB signaling pathways. Moreover, in co-culture the levels of the IL-17 signaling pathway proteins CCL2 and IL-8 were increased in the culture supernatants. The IL-17 signaling pathway is highly involved in the inflammatory process, auto-immune diseases and in the tumor microenvironment [42].

The leukemia cell-induced changes in BMSCs were different than those induced by CD34^+^ cells. The CD34^+^ cells from healthy donors induced changes in 4904 BMSC genes, but the fold change in expression was low. The genes most up-regulated by CD34^+^ cells were *SERPINB2, IL1B, RTP3, CCL7* and *IL8,* and the pathways most represented among the differentially expressed genes were involved with metabolism.

Our gene expression profiling results found some differences in the effects of the three leukemia cell lines on BMSCs: TF-1 and K562 stimulated BMSC pro-inflammatory molecule production, while TF-1α down-regulated BMSC *Col3A1* expression and up-regulating *IRF8* although with a small fold change and the pathways most represented in the differentially expressed genes included Rac, actin cytoskeleton, growth factor hormone and death receptor signaling.

The analysis of BMSC-leukemia cell co-culture supernatant partially confirmed our gene expression data. The factors CCL2, IL-8, IFN-γ and CD40L were detected in the supernatant. We found that the level of CCL2 was the highest in BMSCs co-cultured with TF-1, lower with K562 and the lowest in BMSCs co-cultured with TF-1α. The levels of IFN-γ, CD40L and IL-8 were elevated in the co-culture supernatants; however, the magnitude of the changes in the factor levels differed among the three leukemia cell line experiments confirming their different effects on BMSCs.

We selected the leukemia cell lines according to their phenotype, with TF-1α being closer in phenotype to a leukemia stem cell and our results suggest that BMSCs might react to leukemia cells in a different way than LSCs. The variance in the effects of 3 leukemia cell lines also suggest that differences in the nature of the effects of the leukemia cells on BMSCs might contribute to differences in the clinical presentation among leukemia types. Interestingly, previously published studies of patients with myeloid leukemia and acute lymphocytic leukemia have shown a deregulation of serum cytokine and chemokine profiles including higher levels of CCL2 and IL-8 [43-46] and in myeloid leukemia elevated levels of CCL2 and IL-8 were associated with an unfavorable prognosis [43-45]. Other studies have found that CCL2 and IL-8 inhibit myeloid progenitor proliferation [47-49].

We also noted differences in supernatant factor levels among cultures with BMSCs from different donors. This is likely due to differences among the BMSCs. Our group has previously shown substantial variance among BMSCs from healthy donors [50]. The results of the current study found that the cytokine expression was variable among the assays which used BMSCs from three different donors; BMSCs from only one of the donors reacted to the leukemia cells by increasing the expression of IFNγ and CD40L. Moreover, the levels of CCL2 and IL-8 increased in the BMSCs from all three donors, but by different amounts. We speculate that variances among patients in outcome and response to the treatment might also be ascribable, in part, to differences among their bone marrow stromal cells. Others have also studied BMSC donor variations in cytokines expression profile and have found that the basal and post-inflammatory stimulation cytokine/chemokine profiles are donor-dependent in *in vitro* experiments [51]. Much of the change in BMSCs induced by leukemia cells is likely due to soluble factors secreted by leukemia cells.

In conclusion, our results reveal that BMSCs react to leukemia cells by changing the profile of their expressed cytokines and chemokines to an IL-17 signaling profile. In a microenvironment as finely regulated as the hematopoietic niche, this alteration of secreted factors likely collaborates with leukemia features to create a competitive niche more favorable to leukemia stem cells [52,53].

## Competing interests

The authors declare that they have no competing interests.

## Authors’ contributions

SC, PJ: performed experiments and wrote the manuscript. JR, MS, JJ: generated BMSC. HW and YZ: helped with sample collections. LC: data analysis, FM and DS: study design and edited the draft manuscript. All authors read and approved the final manuscript.
